# Establishing an Inpatient Dual Diagnosis Support Service in Essex: A Quality Improvement Project in Essex Partnership University NHS Foundation Trust

**DOI:** 10.1192/bjo.2026.11507

**Published:** 2026-06-30

**Authors:** Anthony Umeadi

**Affiliations:** Essex Partnership University NHS Foundation Trust, Colchester, United Kingdom

## Abstract

**Aims:**

Co-occurring mental health and substance use disorders are associated with poorer outcomes, prolonged inpatient admissions, frequent readmissions, and fragmented care. Despite national policy advocating integrated treatment, services remain largely siloed, resulting in the persistent “wrong door” phenomenon highlighted in Carol Black’s Independent Review of Drugs. Local needs assessment within Essex Partnership University NHS Foundation Trust (EPUT) identified low staff confidence, lack of clear pathways, minimal psychological input, and poor continuity between inpatient and community services for this population. This Quality Improvement (QI) project aimed to design and implement a pilot inpatient Dual Diagnosis Support Service to improve access to integrated care, enhance staff capability, reduce stigma, and strengthen transition from inpatient admission to community-based support.

**Methods:**

A pilot Dual Diagnosis Liaison Service was implemented across three adult inpatient wards in Colchester using existing workforce capacity and no additional funding. The intervention was co-designed with ward managers, multidisciplinary staff, and patients, incorporating lived-experience priorities. Key components included: routine dual diagnosis assessment within seven days of admission; specialist consultation on integrated treatment planning; motivational enhancement and psychoeducation; liaison with psychology andsocial services; and early, structured signposting to community substance misuse and social support services prior to discharge. A phased QI methodology was adopted, supported by governance oversight and iterative service refinement. Process and outcome measures included staff-reported confidence, access to specialist advice, clarity of discharge pathways, and patient experience feedback. Workforce development was embedded through targeted training and ongoing specialist consultation.

**Results:**

Early qualitative feedback indicates improved staff confidence in managing dual diagnosis presentations and increased clarity regarding referral, discharge planning, and community follow-up. Ward teams reported improved access to specialist expertise and enhanced collaboration with external services. Patients valued timely, non-stigmatising support addressing both mental health and substance use needs, particularly early engagement and psychological input. The pilot demonstrated feasibility, acceptability, and scalability within existing resources, directly addressing previously identified service gaps and aligning with national priorities for integrated care and reduction of avoidable hospitalisation.

**Conclusion:**

This QI project demonstrates that an inpatient Dual Diagnosis Support Service can be implemented effectively without additional funding, delivering early improvements in staff capability, patient experience, and pathway integration. The structured, co-produced, and data-informed approach supports sustainability and scalability. Findings from the six-month pilot will inform refinement and planned expansion into community mental health teams, contributing to system-wide improvements in care for people with co-occurring mental health and substance use disorders.

